# Hidden Semi-Markov Models-Based Visual Perceptual State Recognition for Pilots

**DOI:** 10.3390/s23146418

**Published:** 2023-07-14

**Authors:** Lina Gao, Changyuan Wang, Gongpu Wu

**Affiliations:** 1 Optical Engineering, Xi’an Technological University, Xi’an 710021, China; gaolina@st.xatu.edu.cn (L.G.);; 2 School of Computer Science, Xi’an Technological University, Xi’an 710021, China

**Keywords:** visual perception, state detection, hidden semi-Markov model

## Abstract

Pilots’ loss of situational awareness is one of the human factors affecting aviation safety. Numerous studies have shown that pilot perception errors are one of the main reasons for a lack of situational awareness without a proper system to detect these errors. The main objective of this study is to examine the changes in pilots’ eye movements during various flight tasks from the perspective of visual awareness. The pilot’s gaze rule scanning strategy is mined through cSPADE, while a hidden semi-Markov model-based model is used to detect the pilot’s visuoperceptual state, linking the correlation between the hidden state and time. The performance of the proposed algorithm is then compared with that of the hidden Markov model (HMM), and the more flexible hidden semi-Markov model (HSMM) is shown to have an accuracy of 93.55%.

## 1. Introduction

The cockpit of an aircraft is an integrated and efficient human–machine environment that provides the pilot with the human–machine interface to perceive the aircraft’s overall status and to control the flight in real-time [1]. The cockpit of the modern airplane presents a complex visual scene where the pilot’s field of view is filled with a dense array of instruments and displays that communicate the state of motion and flight parameters regarding the aircraft [2]. As aviation equipment becomes more complex and the number of flights increases, it becomes possible to develop accident prevention concepts based on mathematical and probabilistic models using detailed information on the aircraft’s piloting parameters and the status of the pilot or other crew members. By observing these information displays, the pilot can assess the state of the airplane. As the flight progresses, the information displayed on the instrument panel changes in real-time, resulting in a significant challenge for the pilot to monitor closely.

A pilot’s visual perception involves orienting the eyes, head, and body towards the object of interest, a highly dynamic process in which the eyes constantly scan the environment to sample visual information [3]. During the flight, pilots process information in front of their field of vision in a serial fashion and must learn to observe and scan. If these principles are followed during training, their eyes will become sharper, thus enhancing their flight skills and flying techniques. Eye-movement recordings of pilots can therefore provide a window into the pilot’s processing of information from the cockpit display [4].

A visual scanning strategy for pilots can reveal the cognitive process of human–machine interaction between the pilot and the cockpit. Our research topic is to measure and analyze the gaze scan trajectory, gaze duration, and dwell time of pilots looking at the instruments during the flight to enhance pilot safety. The transitions between flight instruments should highlight differences in gaze scanning strategy based on calculating dwell patterns and generating a transition matrix to perform machine learning to classify the pilot’s visual perception state.

Past research has shown that pilot perception errors are one of the major causes of a lack of situational awareness. Despite extensive research, there is currently a lack of appropriate systems to accurately detect errors in the visual perception state of pilots. The researchers focused more on the impact of visual scanning patterns on pilots’ visual perception during flight operations, and they believed that eye-tracking technology could be integrated with flight simulators to improve pilots’ training efficiency. However, pilot monitoring requires a quantitative analysis of changes in perception degree and the timely detection of changes in pilot status.

Based on the above issues, this paper mines the visual scanning strategies of pilots during flight by fusing data-mining algorithms and HSMM algorithms, specifically using the cSPADE algorithm [5] and using the HSMM algorithm with dwell time to evaluate the perceptual state. What sets our work apart from others is that (1) we construct data rules from correlations of eye-movement measurement trajectories in a given state, quantifying observations under variable quantities as a joint Gaussian distribution to infer the pilot’s hidden state. (2) This study aims to analyze pilot visual gaze perception, discover the knowledge hidden in the gaze trajectory, and address how the information perceived by the pilot from the visual information is correlated and what decision information affects the flight.

The rest of the paper is structured as follows: In the next section, we describe the work related to pilot visual scanning patterns and hidden Markov state detection. Section 3 describes the methodology of the HSMM. Section 4 elaborates on our proposed approach for pilot visual perceptual state identification and presents the experimental structure and analysis. Finally, the conclusion is drawn in Section 5.

## 2. Related Work

### 2.1. Pilot Visual Scanning Strategy

Eye tracking has now become a widely used method in human–computer interaction, scene perception, cognitive neurology, interaction design, human factors engineering, visual search, and other fields [6]. As early as the 1950s, Fitts [7] began a series of studies on the eye-movement characteristics of pilots, where they recorded the eye-movement characteristics of pilots during landing to lay the foundation for the design of aircraft cockpit instrumentation and its importance. A survey by Lefrancois [8] also reported that eye tracking is an effective tool for improving pilot monitoring strategies and that training by learning videos of expert eye gaze patterns can help novice pilots improve their flight gaze strategies. Bellenkes et al. [9] argue that expert gaze durations are shorter and gaze at instruments more frequently. Kasarskis et al. [10] note that expert pilots perform more visual stares, have shorter dwell times, and have more structured modes of visual scanning than novices and that more advanced visual scanning strategies are associated with higher landing performance.

Attention sharing and perception during instrument scanning are two essential elements that pilots need to master. The rules for instrument scanning change with the phase of the flight—the various instruments play a vital role in each stage of the flight. Olivier et al. [11] suggested increasing flight safety by improving the pilot’s monitoring strategy. Highly competent pilots exhibit higher perceptual efficiency and focus most of their time mainly on vital flight instruments.

### 2.2. Application of HSMM

To quantitatively assess the work state and workload of a person or machine, latent variable models are often used to detect the level of cognitive state, the most typical of which is the hidden Markov model [12]. The HMM is a probabilistic model of temporal sequences that describes the generation of state sequences from hidden Markov trains and corresponding observation sequences from state sequences. One or more states are usually associated with each stage to model the process affected by the state transition. XiaoH et al. [13] used the HMM to assess the state of health of mechanical axes and predict state transitions in real-time. XiaoR et al. [14] used the ecological health level as a hidden state and related it to external observations through a hidden Markov model, opening up new research avenues for urban ecosystem health assessment. Wang et al. [15] used the Gaussian mixture–hidden Markov model (G-HMM) to model the oculomotor scan pathway, providing a new approach to the screening, quantification, and assessment of disease. Yunpeng Su et al. [16] used the HMM algorithm to make welding operations easier for robots in mixed-reality situations, reducing the time and cost even more than applications that did not use the HMM.

The hidden semi-Markov model is an extended model of the Markov model with the advantage that each state can have a different state duration [17]. Yang et al. [18] trained machines to process time series to identify state problems using a hidden semi-Markov model, taking into account the dependence of state duration. Kung-Jeng Wang et al. [19] propose a method for Human–Robot Collaboration (HRC) that allows the embedding of a risk-aware module into a hidden semi-Markov model. The specific step is to link assembly rate and risk perception, requiring a reduction in robot assembly cycle time while reducing safety risk. Manoharan et al. [20] consider the relationship between the temporal distribution of states in the HSMM, specifically as applied to outlier detection in large datasets. The artificial HSMM has significantly improved detection accuracy for large multivariate datasets. Among the compression-aware algorithms, Tian Xin et al. [21] proposed a compression-aware target localization algorithm based on the hidden semi-Markov model to achieve both coarse and precise localization processes. The experimental results again verified that the HSMM outperformed the HMM.

In current research applications, most focus on fault diagnosis [22], risk perception [23], intent recognition [24], wear monitoring, and remaining life prediction [25]. This paper provides a solution to the task ambiguity arising from overlapping instruments, mainly using the HSMM. The approach uses information about the temporal sequence of pilots’ instrument gaze to estimate the time course of their attentional transitions between different tracking tasks.

## 3. Model Structure for Pilot State Identification

### 3.1. cSPADE-Based Sequence Pattern Mining

The visual gaze sequence data is the gaze behavior data of pilots looking at the instrument panel during flight. We believe that the process of mining frequent patterns is the process of sequence pattern discovery, and frequent patterns are the main result of sequence pattern discovery, and we construct a rule base of pilot gaze behavior by mining different degrees of sequence patterns. Regarding frequent pattern mining algorithms, the most applicable algorithm for trajectory mining is sequence pattern mining, as the sequence contains temporal information and the duality problem can be solved; for example, a current frequent pattern is B–C–A–D. The order of position sequences in this sequence cannot be broken up. We first mined the gaze patterns at different stages of flight in the collected eye-movement sequence data using the cSPADE algorithm. The exact procedure of the algorithm is shown below (see Algorithm 1). The cSPADE algorithm uses combinatorial properties to divide the original AOI sequence pattern mining into smaller sub-problems that can be solved independently using simple ID-list concatenation operations. The algorithm is thus parallelizable, and in addition, the parallelization is linearly scalable concerning the size of the input database.

In the take-off phase, for example, we construct a rule base by mining the gaze rules for all AOI sequences of the number of flight times by first computing all sequence patterns of length 1 using the scanning algorithm. We then compute all sequence patterns of length two and calculate the number of supported sequences for each pair of items in a two-dimensional matrix. The original vertical representation of the database is first converted to a horizontal translation to calculate the number of supported sequences achieved by another database scan. The subsequent N-length sequence pattern can be formed by concatenating (N + 1) length patterns using their ID list (a list of positions in the sequence for each item).

The support of each item can also be easily calculated from the ID list, as we only need to count the number of different sequences in which it appears. It is also important to note that this method of concatenating ID lists is only valid from 3-length patterns onwards, as ID lists for length 1 and 2 modes can be quite large and may not be suitable for memory. At the end of each round, infrequent sequence patterns ought to be removed, ensuring that only frequent patterns get inflated. In our experiments, we believe that we should look for a pattern with a length of at least 6, as we set the AOI to region 6. The algorithm can be executed using either a breadth-first or depth-first search and ends its execution once there are no more patterns to expand further.
**Algorithm 1.** The cSPADE algorithm$cSPADE\left(min\_sup\right):$ 1. $t=parentclasses\;l_{i};$ 2. $for\;eachparentclassl_{i}\in tdoEnumerate-Frequent\left(l_{i}\right);$ **Enumerate-Frequent(*S*):** 1. $for\;all\;sequences\;O_{i}\in S\;do$ 2.  $if\;(maxgap)//join\;with\;F_{z}$ 3.   $l=Prefix-Item(O_{i});$ 4.   $N=\{all2-sequences\;O_{j}\;inclass\left[l\right]\}$ 5. else//self−join 6.   $N=\{all\;sequences\;O_{j}\in S,with\;j\geq i\}$ 7.  for all sequences m∈D do 8.   $if\;\left(length\left(R\right)<{=max}_{l}\;and\;width\;\left(R\right)<={max}_{w}\right)$ 9.     and accuracy (R)≠100%) 10.     $L\left(R\right)=Constrained-Tcmporal-Join\left(L\left(O_{i}\right)\right),$ 11.          L(a),min_gap,max_gap,window); 12.      $if(\sigma (R\;,C_{i})\geq minsup(C_{i}))\;then$ 13.        $T=TU\left\{R\right\};\;print\;R;$ 14.   Enumerate-Frequent (*T*); 15.  delete *S*;

### 3.2. Pilot Perceptual Behavior Modeling

The critical questions in modeling pilot visual perception in this study are the following: (1) How are the hidden states associated with the observation sequences? (2) What are the conditional probabilities of the observation sequence and the maximum hidden state sequence given the model and the observation sequence? (3) How can model parameters be learned in new input data to improve the pilot’s gaze perception? (4) When does the pilot’s dwell time at each task or each state begin or end? To answer the above questions, we chose HSMM for modeling and optimization.

#### 3.2.1. Visual Perception Based on HSMM Identification of Pilots

General representation of HSMM. In our HSMM, the hidden state represents the pilot’s perceptual level. In contrast, the visible state represents the pilot’s eye-movement gaze sequence. We set the pilot to have three states—high perception (HP), moderate perception (MP), and low perception (LP) sequences (S1, S2, S3)—each of which is associated with a certain level of the task. Each state continuously processes a certain amount of work, and this is represented as the state duration. The duration of each state takes the form of a duration probability density, which is assumed to follow a normal distribution. Each state, $s_{i}$, represents the probability that the state $s_{i}$ has duration $d_{i}$. The state duration is also an indicator of the pilot’s status; a short period for a good state means the pilot is in a stable condition.

During an instrument flight, the pilot must constantly double-check multiple instruments because no single plane instrument can give a complete picture of the aircraft’s behavior. If an overlapping instrument is being scanned simultaneously with other objects belonging to a specific tracking task, then this instrument is more likely to be used for that task. The probability of the conversion of one AOI gaze to another AOI is computed from the pattern conversion matrix, and we use the HSMM algorithm to assess the quality of the pilot’s gaze scan pattern during the various phases of flight.

The hidden semi-Markov model (HSMM) is a derivative of the HMM that adds a time component to the structure of the fully defined hidden Markov model (HMM), overcoming the limitations of the HMM due to the assumption of Markov chains. The most significant difference between the HSMM and the HMM is that the HMM produces one observation per state, whereas the HSMM produces a series of values per state.

In the HSMM, we denote the hidden state at time t by S_t_, and O is a sequence of observed statuses. In the fragment HSMM, there are N states, all hidden and not directly observable. The transitions between states conform to the transition matrix A, and the probability of transition from state i to j is a_ij._ Analogous to the standard HMM, we assume that the state at state moment t is S0 and that the initial distribution of states is π. The transition process S_ql−1_→S_ql_ of the macro-state is consistent with a Markov process.

(1) $$P(s_{ql}=j|S_{ql-1}=i)=a_{ij}$$

The state transition S_t−1_→S_t_ is usually not a Markov process, and this is the reason why the model is called “semi-Markov”. In the semi-Markov case, the transition process in the Markov model only holds when the device transitions from one macro-state to another.

**Figure 1:**
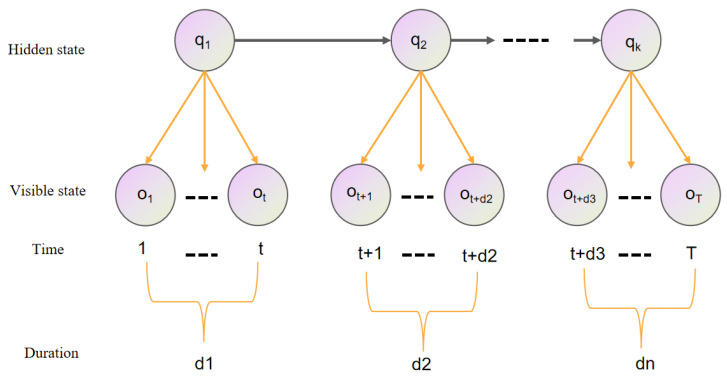
The general HSMM structure.

In terms of parameter setting, the model parameters of the HSMM can be written as $\lambda =\left(\left\{\pi_{i}\right\},\left\{a_{ij}\right\},\left\{b_{i}(l)\right\},\left\{P_{i}(d)\right\}\right)$ (where $\pi_{i}=P\left(q_{1}=s_{i}\right)$ is the probability of the system being in the initial state, satisfying the restriction:$\sum_{i=1}^{N}\pi_{i}=1$). The model structure is shown in Figure 1 and is defined as shown below:

(1) Suppose the hidden-state sequence is a first-order Markov chain:

(2) $$a_{ij}=P\left(q_{t+1}=s_{j}|q_{t}=s_{i}\right)$$

(3) $$\mathrm{and}\;\sum_{j=1}^{N}a_{ij}=1,a_{ij}\geq 0,1\leq i\leq N$$

(2) The probability of an observation occurring in the system, conditional on the state being at moment t, is:

(4) $$b_{i}(l)=P\left(o_{t}=v_{l}|q_{t}=s_{i}\right)$$

(5) $$\mathrm{and}\;\sum_{l=1}^{M}b_{i}(l)=1,1\leq i\leq N$$

(3) The probability that the system resides in state Si at moment t for time d is:

(6) $$P_{i}(d)=P(\tau_{t}=d|q_{t}=s_{i})$$

(7) $$\mathrm{and}\;\sum_{d=1}^{D}P_{i}(d)=1,1\leq i\leq N,\tau_{t}=d$$

Time $q_{t}=s_{i}$ is the time that the system has been resident in state *S_i_* up to time t. D is the maximum possible residence time in any state.

The HSMM framework consists of two layers of stochastic processes. One is a hidden-state process, which is not directly visible but is assumed to follow the first-order Markov process transfer rules. The other is an observed symbolic process, which is physically observable and has a probability distribution based on the current hidden state. For our study, the scanning gaze quality is modeled as a hidden-state process, with the AOI gaze sequence corresponding to the observation process. It is worth noting that many researchers have calculated transfer probabilities between fixations, which is equivalent to calculating Markov matrices for fixed objects.

In the flight simulation, we asked each pilot to express as many instrument readings or current intentions in words as possible, such as currently 1500 feet, heading left, climbing a little too low, and turning right. These reports show the tracking tasks performed at each moment, i.e., the flight tasks mentioned above, followed by the conversion of the corresponding tasks into training data. If the task matched the verbal description at the reporting time or within 1 s, we considered the description to be a “match” to the gaze estimate. Verbal reports on single-parameter instruments are usually matched, so we chose to omit them from the analysis. We considered reports related to overlapping instruments to be the only ones included in this verification process, and we ultimately classified them as high, medium, or low according to the degree of matching.

The specific process follows as shown below: (1) Parameter training: we construct the HSMM and use the EM algorithm for model training (parameter estimation) to estimate the model parameters including the state transfer probabilities. (2) State duration calculation: through model parameter estimation, the probability density function of each macro state duration can be obtained, from which it is possible to calculate the mean value of the state duration. (3) State identification: we find the state labeling sequence with maximum probability based on model parameters and eye-movement gaze sequence data and then determine the pilot’s visual perception ability using the Viterbi algorithm.

#### 3.2.2. A Forward–Backward Solution Algorithm for the HSMM

It is necessary to solve the evaluation and learning problem of the HSMM before applying the HSMM for visual perceptual state recognition. So, we are given the observations $o_{1},o_{2},\cdots ,o_{t},$ as well as the parameters of the HSMM $\lambda =(\Pi ,A,B,p_{i}(d))$ in the case of tuning the model parameters to maximize the value p(o|λ). The basic algorithm, which addresses the HSMM evaluation and learning problem, follows.

The forward variable is the probability that, given the conditions of the model λ, a partial sequence of observations $o_{1},o_{2},\cdots ,o_{t},$ is produced before t and that the state is $s_{i}$ at t and the time at which the state remains at t.

(8) $$\alpha_{t}(i,d)=P\left(o_{1},o_{2},\cdots ,o_{t},q_{t}=s_{i},\tau_{t}=d|\lambda \right)$$

(9) $$\alpha_{t+1}(j,1)=\sum_{i=1}^{N}\sum_{\tau =1}^{D}\alpha_{t}(i,\tau )a_{ij}b_{j}\left(o_{t+1}\right)P_{j}(1),\;(j\neq i)$$

(10) $$\alpha_{t+1}(j,d+1)=\alpha_{t}(j,d)a_{ij}b_{j}\left(o_{t+1}\right)P_{j}(d+1),\;(j=i)$$

The backward variable is the probability of a partial sequence of observations from time t+1 to the end, given a model λ and a state $s_{i}$ at time t and a residence time *d* in this state.

(11) $$\beta_{t}(i,d)=P\left(o_{t+1},o_{t+2},\cdots ,o_{T}|q_{t}=s_{i},\tau_{t}=d,\lambda \right)$$

Initialized as

(12) $$\beta_{T}(i,d)=1,\;1\leq i\leq N,1\leq d\leq D.$$

The backward variables can be calculated iteratively, as follows:

(13) $$\beta_{t}(i,d)=\sum_{j=1,j\neq i}^{N}a_{ij}b_{j}\left(o_{t+1}\right)P_{j}(1)\beta_{t+1}(j,1)+a_{ii}b_{i}\left(o_{t+1}\right)P_{i}(d+1)\beta_{t+1}(i,d+1),$$

t=T−1,T−2,⋯,1, 1≤i≤N.

Let $r_{i}(i,d)$ be the probability that the state at t is $s_{i}$ and the residence time in this state is d, given the observed sequence O and model λ, i.e.,

(14) $$r_{t}(i,d)=P\left(q_{t}=s_{i},\tau_{t}=d|O,\lambda \right)$$

(15) $$r_{t}(i,d)=\frac{\alpha_{t}(i,d)\beta_{t}(i,d)}{\sum_{i=1}^{N}\sum_{d=1}^{D}\alpha_{t}(i,d)\beta_{t}(i,d)}$$

(16) $$\xi_{t}(i,j,d)=\frac{\alpha_{t}(i,d)a_{ij}b_{j}\left(o_{t+1}\right)P_{j}(1)\beta_{t+1}(j,1)}{\sum_{i=1}^{N}\sum_{j=1}^{N}\sum_{d=1}^{D}\alpha_{t}(i,d)a_{ij}b_{j}\left(o_{t+1}\right)P_{j}(1)\beta_{t+1}(j,1)},i\neq j$$

Let A and B be defined by Equation (12) and in Equation (15) as well as Equation (16), then the model parameters are re-estimated.

(17) $${\bar{\pi }}_{i}=r_{1}(i,1),\;1\leq i\leq N$$

(18) $${\bar{a}}_{ij}=\sum_{i=1}^{T-1}\xi_{t}(i,j,d)/\sum_{i=1}^{T-1}r_{t}(i,d),\;1\leq i\neq j\leq N$$

(19) $${\bar{p}}_{i}(d)=\sum_{i=1}^{T}r_{t}(i,d)/\sum_{t=1}^{T}\sum_{d=1}^{D}r_{t}(i,d),\;1\leq i\leq N$$

(20) $${\bar{b}}_{i}(l)=\sum_{t=1}^{T}r_{t}(i,d)\cdot \delta_{a_{i},v_{l}}/\sum_{i=1}^{T}r_{t}(i,d),\;1\leq i\leq N$$

where, when *o_t_* = *v_t_*, $\delta_{a_{i},v_{l}}$ = 1, otherwise $\delta_{a_{i},v_{l}}$ = 0.

## 4. Experimental Design and Analysis of Results

### 4.1. Experimental Equipment

**Figure 2:**
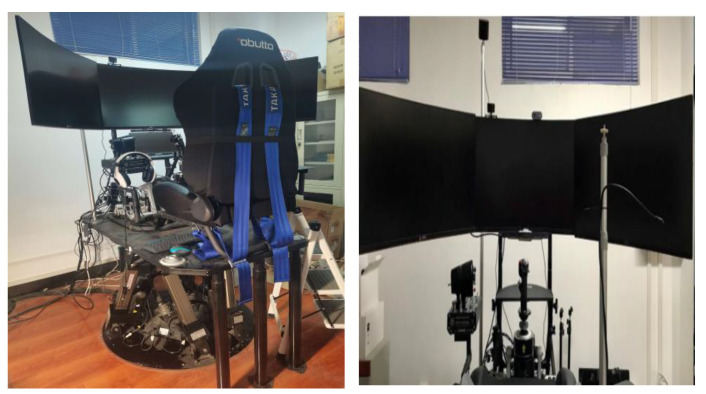
Six-degrees-of-freedom flying motion platform.

The experiment was simulated on a flight simulator with a 6-DOF motion platform structure, where the simulated flight platform includes six-axis motion, a display, a flight rocker, and a data acquisition instrument. This six-degrees-of-freedom flight simulator has a strong sense of realism, with a flight environment, flight attitude, and handling very similar to that of a real aircraft. At the same time, the three LCD monitors stitching together to form a semi-enclosed shape can increase immersion and realism during the flight simulation. The flight experiment platform is shown in Figure 2.

**Figure 3:**
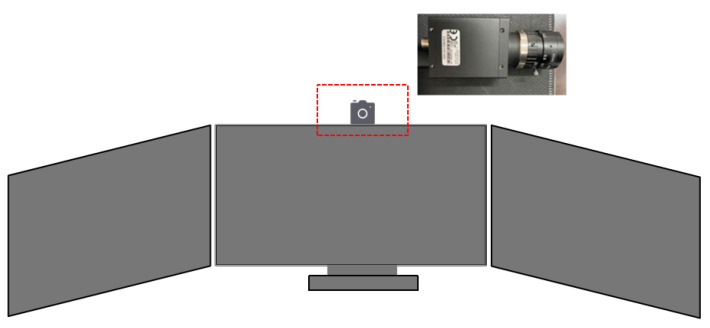
Eye-tracking equipment.

This paper uses a homemade laboratory eye-tracking system to track the user’s bilateral eye movements. The eye-tracking device is placed above the monitor screen and detects eye movements through the reflection of the infrared camera lens in the eye-tracking device on the cornea to achieve the detection of eye movement and then uses a deep learning algorithm to estimate where the user’s eyes fall. This eye-tracking device is highly accurate, has a sampling frequency of 100 HZ, is non-invasive, and does not cause discomfort to the subject performing the task. The device setup is shown in Figure 3.

### 4.2. Task Setting and Data Collection

#### 4.2.1. Task Setting

We used Digital Combat Simulator World software to configure the mission and chose the Su-25T to prepare the pilot for the flight by studying maps and weather information relating to the scheduled time, route, and destination.

**Figure 4:**
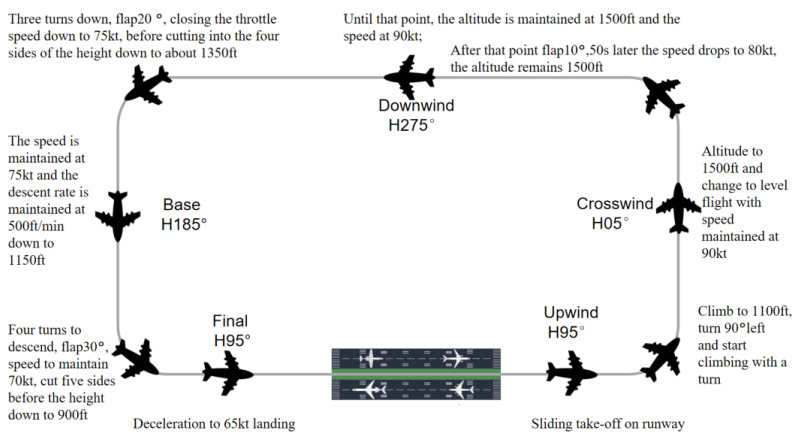
Schematic diagram of the mission.

The experiment was set up as a five-sided flight (airfield traffic pattern), with a left take-off and landing course, meaning that the aircraft was only allowed to turn left in the flight path, and the experiment was conducted in accordance with the specified heading and target altitude. The mission focuses on enhancing the training of pilots in take-off and landing skills and is now widely used in visual flight. It mainly includes take-off and climbs, turns, leveling off, sliding down to land on approach, etc. The main objective is to help the pilot master the flight method and the state control of the flight during the flight. Through constant training in flight handling methods, the process can help participants to adjust their perception strategies. The flight diagram is shown in Figure 4.

During take-off, subjects check the aircraft’s interior and exterior runway for proper functioning. During the take-off phase, the plane proceeds in a straight line and accelerates until it is well above the ground. The subject then retracts the landing gear and wings while checking the aircraft’s climb attitude, speed information, and altitude information.

During the level flight phase, the subject is flying at a constant altitude and speed. During this phase, the pilot follows a fixed heading and checks several flight parameters such as altitude, speed, flight path, and attitude. During the level flight phase, the pilot completes the second, third, and fourth sides. During this phase, the subject also performs tasks such as locking onto the Downwind target and preparing for a return.

During the approach phase, the subject is required to adjust the aircraft’s heading to the runway extension, release the flaps, adjust the angle of descent and throttle, gently bring the stick back and reduce the throttle at 6–7 m above the ground, continuously adjust the aircraft’s sink rate, and lift the nose until the plane is grounded. When the plane is grounded, the subject must maintain the direction of the glide, slowly retract the throttle, gradually brake to reduce speed, and leave the runway.

#### 4.2.2. Data Collection

The experiment recruited 15 flight cadets with an average age of 25 years. They are familiar with the standards and procedures for flight safety performance assessment and have obtained recognition from professional pilots, meeting the requirements of the experiment. They had normal or corrected-to-normal vision. They all signed an informed consent form before the start of the laboratory. In addition, this study has been reviewed by the Ethics Committee of our unit.

During the flight, we used eye-tracking measurements using an eye-tracker to assess the reliability of the eye-tracking device in terms of the accuracy and precision of the data. We consider accuracy to be the average error between the physical position of the eye and the sight location captured by the eye-tracker. Precision is the extent to which the eye tracker continuously records the same gaze point, for instance, through the root mean square measurements of consecutive samples. With proper configuration and calibration, the reliability of eye-tracking devices can meet these requirements. Therefore, setting up and calibrating the device for each participant before the experiment is a process that takes between 1 and 3 min. Furthermore, the application reliability of the eye-tracking device has been proven when acquiring live eye-tracking data in similar driving simulations.

**Figure 5:**
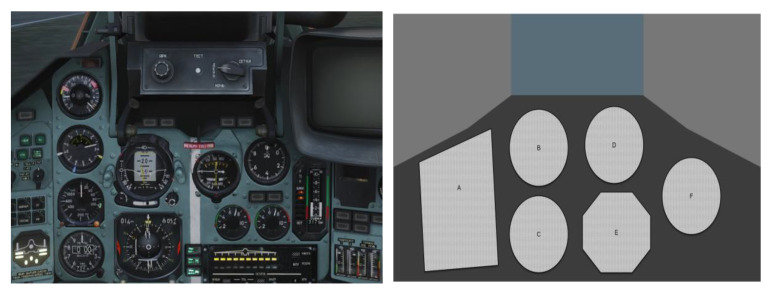
Illustration of the instrument panel area.

The data collection focuses on the pilot’s instrument gaze, and the instrument panel area has been defined into six AOI areas, which are A, Configuration Indicator; B, Airspeed Indicator; C, Altimeter Indicator; D, Attitude Direction Indicator; E, Horizontal Situation Indicator; F, Vertical Velocity Indicator; E, Horizontal Situation Indicator; and F, Vertical Velocity Indicator. This visual monitoring is essential during the flight phases (take-off, approach phase, and landing), where the data displayed on the instruments need to be verified in real-time against the values expected during the flight stages and where the cockpit allows corrective action to be taken in time to ensure optimum safety levels in the event of parameter deflections. The instrument panel area is shown in Figure 5.

This visual detection activity is structured to redirect the pilot’s visual attention from one instrument to another, and human errors such as incorrect flight trajectories or overspeed on landing often occur due to the poor or insufficient monitoring of cockpit instruments. The challenge of this research work is to improve flight safety, especially by integrating eye-tracking and finding solutions for better pilot training in reducing in-flight surveillance errors.

Eye movements are a window into the pilot’s cognitive state, revealing the attentional path taken by the subject through the vision path. For gaze browsing data acquisition, the flight simulator configures the instrument boundary values and saves the instrument boundary and instrument ID in a configuration file for later use. Subjects’ AOI eye-movement sequences are also composed into a directed chain in chronological order. With each gaze point being the subject assigned to an AOI, the raw eye-movement data are a series of dots. To obtain the sequence of gaze dots, we removed all null and outlier values and detected the gaze dots. Then, in order to compress the data, we merged consecutive and duplicate AOIs, resulting in a database of AOI sequences. A threshold of 200 ms to detect gaze points on the AOIs was set, comprising the average of the non-residence times of each AOI plus a standard deviation as

(21) $$\Phi_{\mathrm{threshold}}=\mu_{\mathrm{NDT}}+\sigma_{\mathrm{NDT}}$$

The Visual Behavior Database (VBD) contains the mean non-dwell time, standard deviation, and threshold values calculated for each AOI in seconds.

### 4.3. Analysis of Results

**Figure 6:**
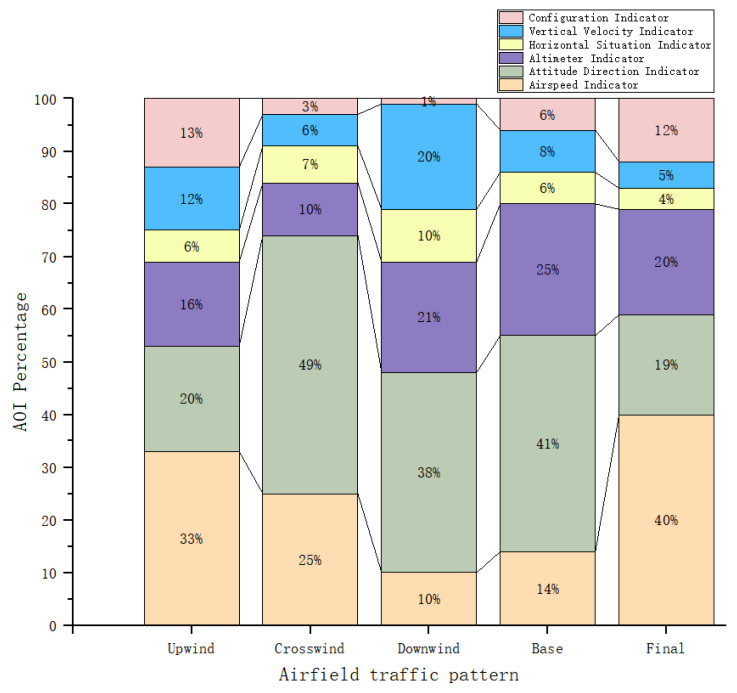
Percentage of flight mission AOI gaze behavior.

We first mined the sequence patterns of different phases of the flight and sorted and filtered them according to the support. We found the highest support for the flight criteria by combining the eye-movement sequences of all testers. The percentage of AOI gaze for different phases of the flight was also analyzed, as shown in Figure 6. We found that the attention to the speedometer decreased and then increased throughout the flight because it takes a lot of time to focus on the speedometer during the takeoff and landing phases, and Upwind and Final are the processes of speed from nothing to something and from something to nothing. In Crosswind, Downwind, and Base missions, pilots pay more attention to the pitch and roll information provided by the attitude meter, so the attitude meter is undoubtedly the most important instrument in these three phases of flight. In Downwind and Base flights, the proportion of attention to the altimeter was high because the aircraft needed to be concerned about whether the altitude of descent reached the approach criteria in Base flights. The proportion of attention to the heading gauge gradually increased and then slowly decreased and the percentage of concern was higher in Downwind flights with relatively long flight times. During the Downwind mission, the flight students paid the highest percentage of attention to the vertical speedometer to ensure altitude and to determine the possibility of stalling in the vertical direction. The mechanical equipment indicator shows the current position of the landing gear, flaps, and deceleration plates, so during Upwind and Final, the focus needs to be on ensuring the aircraft can take off and land properly.

In our experiments, we used three metrics, accuracy (P), recall (R), and F-value, as the metrics for performance evaluation. The metrics are defined as follows:

$$P=\frac{N_{1}}{N_{1}+N_{2}},\;\;R=\frac{N_{1}}{N_{1}+N_{3}}$$

Let us define N_1_ as the number of samples that correctly identify the data, N_2_ as the data misidentified to that state, and N_3_ as the number of instances of samples that belong to that state category but are mistakenly identified to other classes.

Comprehensive evaluation indicators are defined as:

$$F=\frac{(\beta^{2}+1)\times P\times R}{\beta^{2}\times P+R}$$

**Figure 7:**
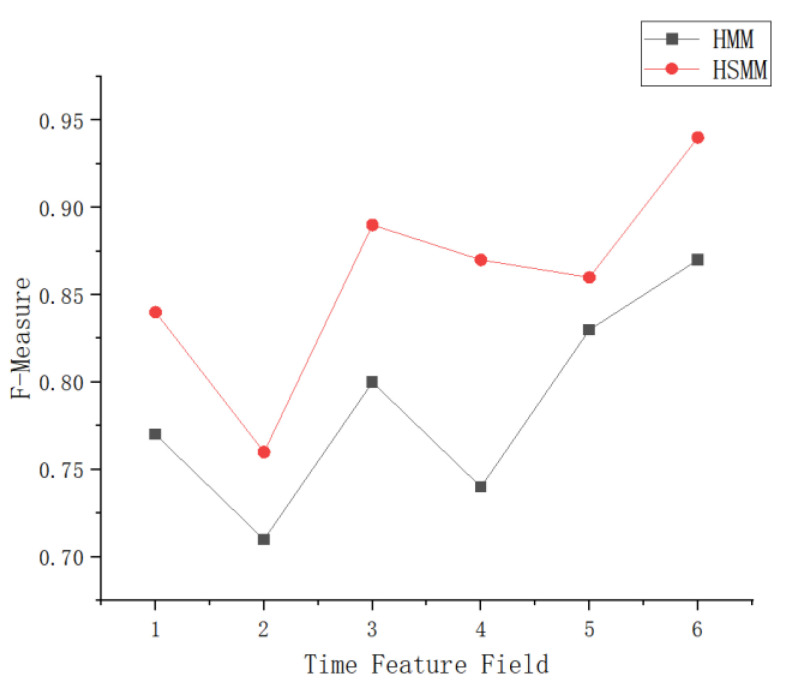
Comparison of HSMM and HMM results.

In this case, the parameter β serves for assigning different weights to the accuracy P and the recall R. We take β = 1 in our experiments, and the accuracy and the callback rate are given the same weight. Meanwhile, we evaluate the HSMM and the HMM separately using the comprehensive evaluation index F. The results are shown in Figure 7. The accuracy and recall for each eigenvalue state and the test results are shown in Table 1. The above test results reflect that the performance of each feature of the experimental system is average when using the HMM for feature extraction, whereas the performance of each topic of the test performed better after the implicit semi-Markov statistical model was introduced. This shows that the HSMM better reflects the generalized situation of eye-movement information and is more suitable for describing practical problems.

## 5. Discussion

In this study, we conducted an identification of pilot visual perception states based on the implicit semi-Markov model (HSMM). By exploring the changes in the pilot’s eye movements during different missions, we have conducted an in-depth study on the problem of the pilot’s loss of situational awareness from the perspective of visual perception. With the help of the cSPADE method to mine the pilot’s gaze rule scanning strategy, we proposed an algorithm based on the HSMM to detect the pilot’s visual perception state and successfully relate the correlation between hidden state and time.

By comparison with the hidden Markov model (HMM), we found that the proposed HSMM algorithm exhibited a higher accuracy of 93.55% in pilot visual perceptual state identification. This shows that the flexibility of the HSMM and its ability to model temporal correlation makes it a better performer in solving pilot perception error problems.

The results of this study have significant practical applications. By accurately identifying the pilot’s visual perceptual state, we can detect and correct the pilot’s perceptual errors promptly, thus improving the pilot’s situational awareness and reducing the impact of human factors on aviation safety. In addition, our study also provides valuable references for further improvement of pilot training and monitoring systems.

However, this study has some potential limitations and directions for improvement. First, our study mainly focused on the relationship between pilots’ eye movements and visual perception states without considering other factors that might influence situational awareness. Therefore, future research can further explore the fusion of multiple sources of information to improve the integrated capability of the pilot’s situational awareness. In addition, our study can be extended to a wider range of flight missions and different types of pilot groups to validate and extend the applicability and reliability of the proposed HSMM.

In summary, this study effectively solves the problem of pilots’ loss of situational awareness through an implicit semi-Markov model-based pilot visual perception state identification method. Future research can further improve and extend this method to promote aviation safety improvement and pilots’ professional competence development.

## Figures and Tables

**Table 1:** Test results and performance index statistics of the models for feature extraction experiments.

	*N*_1_	*N*_2_	*N*_3_	P/%	R/%
HMM	72	18	15	80.00	82.76
HSMM	89	10	9	93.55	91.58

## Data Availability

Not applicable.

## References

[1] Kearney P., Li W.-C., Lin J.J. (2016). The impact of alerting design on air traffic controllers’ response to conflict detection and resolution. Int. J. Ind. Ergon..

[2] Allsop J., Gray R. (2014). Flying under pressure: Effects of anxiety on attention and gaze behavior in aviation. J. Appl. Res. Mem. Cogn..

[3] Martinez-Marquez D., Pingali S., Panuwatwanich K., Stewart R.A., Mohamed S. (2021). Application of eye tracking technology in aviation, maritime, and construction industries: A systematic review. Sensors.

[4] Naeeri S., Kang Z., Mandal S., Kim K. (2021). Multimodal Analysis of Eye Movements and Fatigue in a Simulated Glass Cockpit Environment. Aerospace.

[5] Zaki M.J. Sequence mining in categorical domains: Incorporating constraints. Proceedings of the Ninth International Conference on Information and Knowledge Management.

[6] Sullivan J., Yang J.H., Day M., Kennedy Q. (2011). Training simulation for helicopter navigation by characterizing visual scan patterns. Aviat. Space Environ. Med..

[7] Fitts P.M., Jones R.E. (1949). Eye Fixation of Aircraft Pilots, III. Frequency, Duration, and Sequence Fixations When Flying Air Force Ground Controlled Approach System.

[8] Lefrancois O., Matton N., Gourinat Y., Peysakhovich V., Causse M. The role of Pilots’ Monitoring Strategies in Flight Performance. Proceedings of the EAAP32, European Association for Aviation Psychology Conference.

[9] Bellenkes A.H., Wickens C.D., Kramer A. (1997). Visual scanning and pilot expertise: The role of attentional flexibility and mental model development. Aviat. Space Environ. Med..

[10] Kasarskis P., Stehwien J., Hickox J., Aretz A., Wickens C. Comparison of expert and novice scan behaviors during VFR flight. Proceedings of the 11th International Symposium on Aviation Psychology.

[11] Lefrançois O., Matton N., Causse M. (2021). Improving Airline Pilots’ Visual Scanning and Manual Flight Performance through Training on Skilled Eye Gaze Strategies. Safety.

[12] Fox E.B., Sudderth E.B., Jordan M.I., Willsky A.S. An HDPHMM for systems with state persistence. Proceedings of the 25th International Conference on Machine Learning.

[13] Xiao H., Zeng H., Jiang W., Zhou Y., Tu X. (2022). HMM-TCN-based health assessment and state prediction for robot mechanical axis. Int. J. Intell. Syst..

[14] Xiao R., Yu X., Shi R., Zhang Z., Yu W., Li Y., Chen G., Gao J. (2019). Ecosystem health monitoring in the Shanghai-Hangzhou Bay Metropolitan Area: A hidden Markov modeling approach. Environ. Int..

[15] Wang X., Geng X., Wang J., Tamura S. (2021). A comparative research on g-hmm and tss technologies for eye movement tracking analysis. An enhanced Hidden Semi-Markov model for anics in Medicine and Biology. J. Mech. Med. Biol..

[16] Su Y., Lloyd L., Chen X., Chase J.G. (2023). Latency mitigation using applied HMMs for mixed reality-enhanced intuitive teleoperation in intelligent robotic welding. Int. J. Adv. Manuf. Technol..

[17] Zhu K., Liu T. (2017). Online Tool Wear Monitoring via Hidden Semi-Markov Model with Dependent Durations. IEEE Trans. Ind. Inform..

[18] Yang W., Chen L. (2021). Machine condition recognition via hidden semi-Markov model. Comput. Ind. Eng..

[19] Wang K.J., Lin C.J., Tadesse A.A., Woldegiorgis B.H. (2023). Modeling of human–robot collaboration for flexible assembly—A hidden semi-Markov-based simulation approach. Int. J. Adv. Manuf. Technol..

[20] Manoharan G. (2022). Sivaoutlier detection in multivariate datasets. J. Intell. Fuzzy Syst..

[21] Tian X., Wei G., Wang J. (2022). Target Location Method Based on Compressed Sensing in Hidden Semi Markov Model. Electronics.

[22] Khaleghei A., Makis V. (2015). Model parameter estimation and residual life prediction for a partially observable failing system. Nav. Res. Logist. (NRL).

[23] Li F., Zheng W.X., Xu S. (2021). Stabilization of discrete-time hidden semi-Markov jump singularly perturbed systems with partially known emission probabilities. IEEE Trans. Autom. Control..

[24] Liu Q., Xu S., Lu C., Yao H., Chen H. (2020). Early Recognition of Driving Intention for Lane Change Based on Recurrent Hidden Semi-Markov Model. IEEE Trans. Veh. Technol..

[25] Lin M., Wanqing S., Chen D., Zio E. (2022). Evolving Connectionist System and Hidden Semi-Markov Model for Learning-Based Tool Wear Monitoring and Remaining Useful Life Prediction. IEEE Access.

